# Autoantibodies against N-terminal EF-hand calcium-binding protein 1 in neurological syndromes and cancer

**DOI:** 10.1007/s00415-026-14257-3

**Published:** 2026-11-14

**Authors:** Madeleine Scharf, Ramona Miske, Le Duy Do, Juan Granja Lopez, Kathrin Borowski, Yvonne Müller, Christiane Radzimski, Henning Bagelmann, Monika Baloun, Lars Komorowski, Anne-Laurie Pinto, Géraldine Picard, Jerome Honnorat

**Affiliations:** 1 Institute for Experimental Immunology, Affiliated with EUROIMMUN Medizinische Labordiagnostika AG, Seekamp 31, 23560 Luebeck, Germany; 2 French Reference Center on Paraneoplastic Neurological Syndromes and Autoimmune Encephalitis, Hospices Civils de Lyon, Hôpital Neurologique, Bron, France; 3 MeLiS - UCBL - CNRS UMR 5284 - INSERM U1314, Université de Lyon, Université Claude Bernard Lyon 1, Lyon, France; 4 Clinical Immunological Laboratory Prof. h.c. (RCH) Dr. med. Winfried Stöcker, Groß Grönau, Germany; 5 Kreiskrankenhaus Freiberg, Freiberg, Germany; 6 Present Address: Neurology Department and Stroke Center, Laboratory of Neurological and Cerebrovascular Sciences, Hospital La Paz Institute for Health Research Institute-IdiPAZ (La Paz University Hospital-Universidad Autónoma de Madrid), Madrid, Spain

**Keywords:** Autoimmune, Neurological syndrome, Breast cancer, Lung cancer, Autoantibodies, N-terminal EF-hand calcium-binding protein 1 (NECAB1)

## Abstract

**Background:**

Paraneoplastic neurological syndromes (PNS) are immune-mediated disorders associated with cancer and often precede tumor diagnosis. Identification of novel neural autoantigens may facilitate diagnosis and treatment.

**Methods:**

Sera or cerebrospinal fluid (CSF) from six patients showing a similar uncharacterized immunofluorescence staining pattern on rat hippocampal sections were collected. The target antigen was identified by immunoprecipitation and mass spectrometry and validated using established immunoassays.

**Results:**

N-terminal EF-hand calcium-binding protein 1 (NECAB1) was revealed as target of the autoantibodies. Antigen identification was validated by competitive tissue IIFA, CBA, Western blot and line blot with the recombinant antigen. No reactivity was observed in CBA and line blot with 100 healthy controls or CBA with 303 disease controls. All five clinically characterized patients had breast or lung cancer and presented with limbic encephalitis (*n* = 3), cerebellar ataxia with cognitive decline (*n* = 1), or sensory-motor neuronopathy (*n* = 1).

**Conclusions:**

NECAB1 represents a novel autoantibody target in autoimmune neurological syndromes associated with lung or breast cancer. Samples from patients with suspected neurological autoimmune disorder showing the specific hippocampal interneuron IIFA pattern should be analyzed for NECAB1 autoantibodies by CBA or line blot and cancer screening should be considered.

**Supplementary Information:**

The online version contains supplementary material available at https://doi.org/10.1007/s00415-026-14257-3.

## Introduction

Autoimmune neurological diseases are conditions where neuronal structures of the body are attacked by the immune system. Triggers for the development of autoimmune responses can be cancers, infections or therapeutics like immune checkpoint inhibitors [1, 2]. In paraneoplastic neurological syndromes (PNS) neuronal antigens are expressed ectopically in tumors outside the brain, which is thought to induce the production of autoantibodies against these proteins. Diagnostically, these autoantibodies can function as markers for distinct neurological syndromes as well as for the underlying tumors. Some neuronal autoantibodies show a strong association with specific cancer types and characteristic neurological symptoms, with only a few noted exceptions. Examples include anti-Yo antibodies, which cause paraneoplastic cerebellar degeneration typically linked to pelvic or breast tumors [3], and anti-RGS8 antibodies, which are associated with paraneoplastic cerebellar syndrome in the context of lymphoma [4, 5]. Other antibodies appear restricted to a particular tumor type and neurological syndrome at the time of discovery, such as anti-KLHL11 associated with brain-stem and cerebellar symptoms [6], but later prove to be associated with more diverse clinical manifestations such as seizures, neuropsychiatric dysfunction, myeloneuropathy, and cervical amyotrophy [7]. Anti-Hu antibodies have been recognized in paraneoplastic neurological syndromes for more than 30 years, and their clinical phenotype encompasses a wide range of neurological presentations, including encephalitis, predominantly peripheral neuropathy, and multifocal neurological involvement [8]. Another example of an autoantibody with a broad neurological spectrum is anti-CV2/CRMP5, which has been linked to cerebellar ataxia, sensory-motor neuropathy, retinopathy, and encephalomyelitis [9].

Here, we report on NECAB1 as a novel autoimmune target antigen in patients with neuronal autoimmune diseases and cancer.

## Methods

Reagents were obtained from Merck, Darmstadt, Germany, or Sigma-Aldrich, Heidelberg, Germany, if not specified otherwise.

### Patients

The Clinical Immunological Laboratory Prof. Dr. med. W. Stöcker, Groß Grönau (Germany) and the French Reference Centre for Paraneoplastic Neurological Syndromes and Autoimmune Encephalitis, Hospices Civils de Lyon, Lyon (France), received the samples for the purpose of autoantibody testing between 2019 and 2024 (Groß Grönau *n* = 237,259, Lyon *n* = 62,530). 205 samples from patients with high suspicion for a neurological autoimmune disease were collected by the French Reference Centre. The 205 samples in the screening cohort were obtained from patients with a clinically based high suspicion of a neurological autoimmune disease. All samples were tested negative for established paraneoplastic or encephalitis autoantibodies such as Hu, Yo, CV2/CRMP5, LGI1, CASPR2, and NMDAR, among others. The presence of an underlying tumor was not an inclusion criterion for this cohort. Subsequently, the samples were anonymized and provided to the authors. Written informed consent to the publication of all clinical information was obtained from all patients whose detailed clinical data are reported in this study. An approval from the French Reference Centre for Paraneoplastic Neurological Syndromes and Autoimmune Encephalitis, Hospices Civils de Lyon institutional review board was received (number: 22–748).

The analyzed control panels included sera from 100 healthy donors (healthy controls, HC) and 303 sera or CSF samples from patients with different diseases (disease cohort, DC), including chronic inflammatory demyelinating polyneuropathy (CIDP, *n* = 33 sera), diabetic neuropathy (*n* = 30 sera), Guillain–Barré syndrome (GBS, *n* = 30 sera), small cell lung cancer (SCLC) without PNS (*n* = 49 sera), Alzheimer disease (*n* = 89 CSF), normal pressure hydrocephalus (*n* = 15 CSF), psychosis (*n* = 20 CSF), dementia with Lewy bodies (*n* = 12 CSF), and frontotemporal dementia (*n* = 25 CSF). Patient’s PNS care score was calculated according to the updated diagnostic criteria for paraneoplastic neurological syndromes [10] by summing phenotype points and cancer points, while no antibody points were added. In summary, a maximum of 10 points can be given for a patient, with a score ≥ 8 leading to the diagnosis of definite PNS, while 4–5 points is defined as possible PNS and 6–7 points as probable PNS.

### Indirect immunofluorescence assays

Indirect immunofluorescence assays (IIFAs) were conducted using microscopy slides mounted with a biochip array consisting of brain tissue cryosections (rat hippocampus, rat or primate cerebellum, and murine encephalon) or recombinant HEK293 cells separately expressing 35 brain antigens (neuronal ELAV-like proteins [Hu], cerebellar degeneration-related protein 2 [Yo], NOVA proteins [Ri], collapsin response mediator protein 5 [CRMP5/CV2], paraneoplastic antigen Ma2 [PNMA2], inositol 1,4,5-trisphosphate receptor type 1 [ITPR1], homer protein homolog 3 [Homer3], carbonic anhydrase-related protein VIII [CARP VIII], Rho GTPase-activating protein 26 [ARHGAP26], zinc finger protein of the cerebellum 4 [ZIC4], Delta/Notch-like EGF-related receptor [DNER]/Tr, glutamic acid decarboxylase 65 [GAD65], recoverin, gamma-aminobutyric acid receptor type B [GABAB-R], glycine receptor [GlyR], dipeptidyl-peptidase-like protein 6 [DPPX], immunoglobulin-like cell adhesion molecule 5 [IgLON5], N-methyl-D-aspartate receptor [NMDAR], α-amino-3-hydroxy-5-methyl-4-isoxazolepropionic acid receptor [AMPAR], metabotropic glutamate receptor 1 [mGluR1], metabotropic glutamate receptor 5 [mGluR5], glutamate receptor delta-2 subunit [GluD2], leucine-rich glioma-inactivated protein 1 [LGI1], contactin-associated protein-like 2 [CASPR2], aquaporin-4 [AQP4], myelin oligodendrocyte glycoprotein [MOG], ATPase Na+/K + transporting subunit alpha 3 [ATP1A3], neurochondrin [NCDN], flotillin-1 and flotillin-2 [flotillin1/2], potassium voltage-gated channel subfamily A member 2 [KCNA2], adaptor-related protein complex 3 beta 2 subunit [AP3B2], seizure-related 6 homolog-like protein 2 [Sez6L2], contactin-1 [CNTN1], neurofascin 155 [NF155], and neurofascin 186 [NF186]), recombinant acetone-fixed HEK293 cells expressing either histidine-tagged human NECAB1 or empty vector-transfected HEK293 cells as control. Each BIOCHIP MOSAIC (EUROIMMUN, Lübeck, Germany) was incubated with 70 µL of PBS-diluted serum or CSF at room temperature for 30 min, washed with PBS-Tween, and immersed in PBS-Tween for 5 min. In a second step, either Alexa Fluor 488-labeled goat anti-human IgG (Jackson Research, Suffolk, United Kingdom) or fluorescein isothiocyanate (FITC)-labeled goat anti-human IgG (EUROIMMUN, Germany) were applied and incubated at room temperature for 30 min. If required, nucleic acid stain (TO-PRO™−3 Iodide, Fisher Scientific, Waltham, USA) was added during the second incubation step. Slides were washed again with a flush of PBS-Tween and then immersed in PBS-Tween for 5 min. Slides were embedded in PBS-buffered, DABCO-containing glycerol (approximately 20 µL per field) and examined by fluorescence microscopy. Samples were classified as positive or negative based on the fluorescence intensity of the transfected cells in direct comparison with non-transfected cells and control samples. For the newly developed prototypic NECAB1 CBA the initial sample dilution was determined to be 1:10 for CSF and 1:100 for serum. Endpoint titers refer to the highest dilution showing visible fluorescence.

For competitive immunofluorescence assays 1:320 diluted patient sample (P1) was preincubated with 1:5 diluted HEK293 cell extracts containing the overexpressed recombinant antigen or with empty vector transfected HEK293 control extracts for 1 h prior to tissue incubation.

Results were evaluated by two independent observers using a EUROStar II microscope (EUROIMMUN, Germany) or an LSM700 (Zeiss, Jena, Germany).

### Immunoprecipitation

Hippocampi from rats were dissected and shock-frozen in liquid nitrogen. The tissues were homogenized at 4 °C in solubilization buffer (100 mmol/L tris–HCl pH 7.4, 150 mmol/L sodium chloride, 2.5 mmol/L ethylenediamine tetraacetic acid, 0.5% (w/v) sodium deoxycholate, 1% (w/v) Triton X-100) containing protease inhibitors (Complete mini, Roche Diagnostics, Penzberg, Germany). Insoluble material was sedimented by centrifugation at 21,000 × g at 4 °C for 15 min. For immunoprecipitation, 500 µL of the supernatant were incubated overnight at 4 °C with 15 µL of patient sera and then with Protein G Dynabeads (ThermoFisher Scientific, Dreieich, Germany) for another 3 h to capture immunocomplexes. Beads were washed 3 times with PBS, and eluted with NuPage LDS sample buffer (ThermoFisher Scientific, Schwerte, Germany) containing 25 mmol/L dithiothreitol at 70 °C for 10 min. Carbamidomethylation with 59 mM iodoacetamide (Bio-Rad, Hamburg, Germany) was performed prior to SDS-PAGE (NuPAGE, ThermoFisher Scientific, Schwerte, Germany). Separated proteins were visualized with Coomassie Brillant Blue (G-250) (Merck), and identified by mass spectrometric analysis as described elsewhere [11].

### Recombinant expression of NECAB1 in HEK293 and *E. coli* cells

The cDNA encoding human NECAB1 was obtained from Source BioScience Ltd, Nottingham, UK (clone IRATp970B0288D, BC068448). The coding sequences were amplified by PCR using the template cDNA and DNA oligonucleotide primers (sense NECAB1: ATACGTCTCACATGGAAGATTCCCAGGAGACATCG, asense NECAB1: TATCGTCTCGTCGAGGTTGTTCAGGATCCACCACG, and asense NECAB1-Stop: TATCGTCTCGTCGAGCTAGTTGTTCAGGATCCACCACG). The amplification products were digested with Esp3I (New England Biolabs, catalog number R0734) and ligated with linearized pTriEx-1 or pET24d-N (Merck, Darmstadt, Germany).

His-NECAB1 was transiently expressed in the human cell line HEK293 following PEI-MAX™ mediated transfection (Polysciences, catalog number 24765) according to the manufacturer’s instructions. For IIFA, cells were grown on cover slides and acetone-fixed two days after transfection. To produce recombinant cell lysates, cells were harvested 5 days after transfection and lysed by shear-stress. The lysates were stored in aliquots at − 80 °C until further use. For bacterial antigen production His-tagged human NECAB1 was cloned into the expression vector pET24d. Overexpressed His-NECAB1 was enriched from *E.coli* cells by immobilized metal ion affinity chromatography combined with anion exchange chromatography.

### Immunoblot

Hippocampus lysate or recombinant His-NECAB1 purified from *E.coli* were subjected to western blotting as previously described [4]. Membranes were incubated with sera (dilution 1:200) or mouse anti-His antibody (1:2000, Merck, 70796-3) in 1:5 diluted sample buffer (EUROIMMUN, Germany) for 3 h, followed by incubation with anti-human-IgG-AP (1:10, EUROIMMUN, Germany) or anti-mouse IgG-AP (1:2000, Sigma-Aldrich).

Alternatively, purified His-NECAB1 was coated as a line onto nitrocellulose membrane, and strips were incubated with sera (1:100 dilution) in sample buffer (EUROIMMUN, Germany) for 30 min. Serum incubation was followed by three washing steps with wash buffer (EUROIMMUN, Germany), a second incubation for 30 min with alkaline phosphatase-labeled anti-human IgG (1:10, EUROIMMUN, Germany), three washing steps, and staining with nitro blue tetrazolium/5-bromo-4-chloro-3-indolyl-phosphate substrate (EUROIMMUN, Germany) for 10 min. Dried line blot strips were evaluated by the use of EUROLineScan software (EUROIMMUN, Germany). Signal intensities from 0–5 are rated negative, 6–10 borderline, ≥ 11 positive. Values > 50 are interpreted as strong positive.

## Results

### Clinical and paraclinical features

All patients (*n* = 6) were female, aged between 50 and 84 years at symptom onset. All patients with clinical information available (*n* = 5) suffered from cancer (3 breast cancer, 2 lung cancer) (Table 1). Neurological syndromes included limbic encephalitis (3 cases), cerebellar ataxia with cognitive decline (1 case) and sensory-motor neuronopathy (1 case). All patients with detailed clinical information available (*n* = 4) had a subacute onset of clinical symptoms, and all 5 CSF studied were inflammatory. Different types of immunotherapy, such as methylprednisolone, intravenous immunoglobulin (IVIG) or cyclophosphamide (CP), led to mild improvements of neurological symptoms in three of four cases. In the other case, despite immunotherapy with methylprednisolone, the mRS score was stable over a period of 4 months and the patient passed away due to oncological complications. According to the updated diagnostic criteria for paraneoplastic neurological syndromes [10], all five patients with clinical information were rated with six (patient 4) to seven (patients 1, 2, 5, 6) points, leading to the diagnostic level of probable PNS. Following these criteria, updated in 2021, points are given for each patient on the clinical level, the laboratory level (meaning the autoantibody and its cancer association percentage by literature), and cancer. A maximum of 10 points can be achieved per patient in total. A score of 6–7 points on the scale indicates probable PNS. It must be noted that no points were added at the laboratory level since anti-NECAB1 antibodies, representing a new diagnostic marker, are not yet classified. Considering only the cohort of patients with antibodies against NECAB1 described in this study, 100% of cases with a cancer association would be classified as a high-risk antibody, resulting in the addition of 3 points per patient at the laboratory level, theoretically leading to the diagnosis of definite PNS (≥ 8 points). Detailed description of the patients’ clinical course can be found in the Appendix. Information about time of sample collection in relation to symptom onset and tumor detection are given in Online Resource 2, Tab 2.

**Table 1:** Clinical and paraclinical findings, therapy and outcome of 5 patients with anti-NECAB1 autoantibodies

Patient #, sex, age at neurological onset	Main clinical features	Cancer	Cancer therapy	NECAB1 IgG CBA	Brain MRI (weeks after symptom onset)	CSF white cells per mm3/protein (g/L)/OCB	Immunotherapy	Outcome: mRS (length follow-up); death
#1 female, 53 y	Limbic encephalitis	Breast cancer	N/A	Serum 1:3.200, CSF 1:1.000	N/A	N/A	N/A	N/A
#2 female, 65 y	Cerebellar syndrome with cognitive decline (ataxia, dysarthria, double vision, vertigo)	Breast cancer	HT (letrozole) and TT (abemaciclib)	Serum 1:10.000, CSF 1:320	MRI (13): normal; MRI (36): meningeal irritation	25/1.2/+	Methylprednisolone 1 g/d for 5 days, RTX 3 × 1000 mg	lmprovement of neurological symptoms under immunotherapy^a^, stabilization (38 months)
#4 female, 62 y	Sensory-motor neuronopathy (dysphagia, unintentional weight loss, paresthesia and equilibrium deficits)	Lung cancer (metastatic pulmonary adenocarcinoma, including a right parietooccipital brain metastasis)	CT (carboplatin, taxol)	Serum 1:320, CSF 1:320	MRI (71): right parietal lobe metastasis/ENG: sensorimotor demyelinating neuropathy	12/0.81/+^b^	Methylprednisolone 120 mg/day with progressive tapering over following weeks (18 days)	Peak: 3 Last follow-up: 3 (4 months) Death: peritoneal carcinomatosis and abdominal sepsis
#5 female, 84 y	Limbic encephalitis (memory disturbance, anorexia, weight loss, apathy, headache, pruritus, sleepiness)	Lung cancer (non keratinizing epidermoid cancer with PDL-1 expression; 2 lymph node metastasis)	SURG, CT (carboplatin and taxol), RT	Serum 1:32.000, CSF 1:3.200	MRI (2): bilateral temporal FLAIR hyperintensity. right pontocerebellar angle meningioma	16/0.97/NA	IVIG 200 g over 5 days monthly for 4 months, RTX (125 days)	Peak: 3 Last follow-up: 2 (69 months)
#6 female, 50 y	Limbic encephalitis (generalized seizures, visual and auditive hallucinations, persecutory delusions, memory and executive disturbances)	Breast cancer (IDC grade III Ro+, Ki 50%; 5 mm solitary lung nodule)	Treatment: SURG, CT, RT, HT (anastrozole)	Serum N/A, CSF: 1:100	MRI (N/A): bilateral temporal FLAIR hyperintensity (right > left)	36/0.28/+	6 cycles of CP (750 mg/m2 monthly pulses)	Peak: 3 Last follow-up: 2 (41 months)

For patient 3, no clinical information is available

*CP* cyclophosphamide, *CSF* cerebrospinal fluid, *CT* chemotherapy, *ENG* electroneurogram, *HT* hormonal therapy, *TT* targeted therapy, *IDC* invasive ductal carcinoma, *IVIG* intravenous immunoglobulin, *mRS* modified Rankin Scale, *NA* not available, *OCB* oligoclonal bands, *Peak* modified Rankin Scale at peak of disease, *RT* radiotherapy, *RTX* rituximab, *SURG* surgery

^a^mRS score not determined

^b^Type 4 (mirrored) oligoclonal bands (identical serum and CSF pattern)

### Patient autoantibodies show a distinct pattern in tissue IIFA

**Fig. 1:**
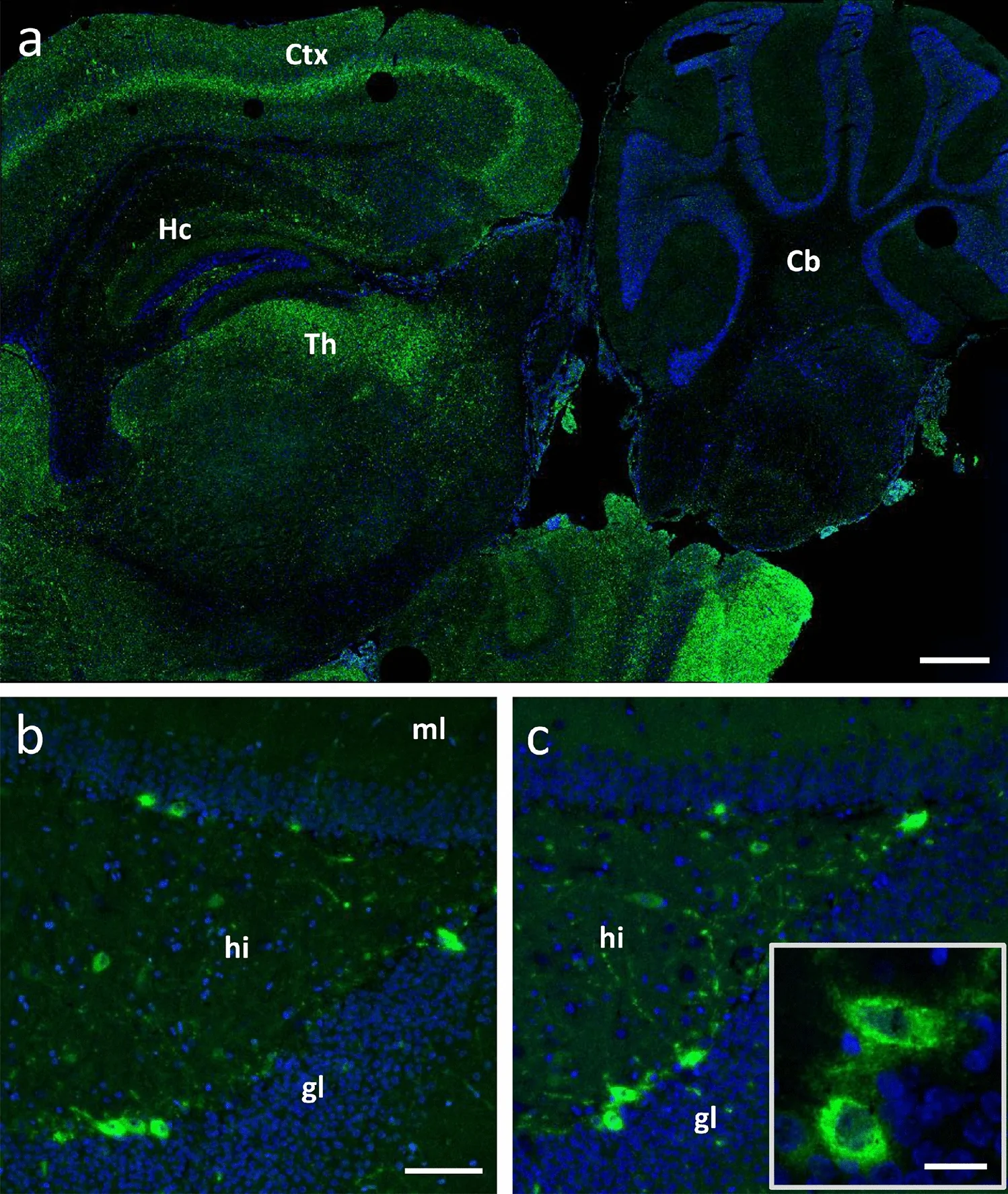
Immunofluorescence assay with rodent brain cryosections. 1:320 diluted patient serum (**a**, **b**) or 1:100 diluted CSF (**c**) of patient 1 was incubated on mouse whole brain (**a**) or rat hippocampal (**b**, **c**) cryosections. In a second step, Alexa Fluor 488 labeled goat anti-human IgG was incubated. Cell nuclei were stained by DAPI (**a**) or TO-PRO-3 Iodide (**b**, **c**). Inlay shows cytoplasmic staining of reactive neurons in the hilus. Scale bars (**a**): 500 µm, (**b** and **c**): 100 µm, insert (**c**): 20 µm. *Ctx* cortex, *Hc* hippocampus, *Th* thalamus, *Cb* cerebellum, *hi* hilus, *gl* granula cell layer, *ml* molecular layer

IIFA with rat brain and serum or CSF samples from index patients (patients 1–3) produced a similar autoantibody binding pattern with cytoplasmic staining of a subset of interneurons located at the border between the granular cell layer and the polymorphic layer (hilus) of the dentate gyrus (Fig. 1). Further somato-dendritic staining is detected in subsets of neurons at the border between stratum lacunosum moleculare and stratum radiatum, layer IV of the cortex, thalamus and hypothalamus, while no staining of cerebellar structures was detected. Sera and CSF samples from patients 4–7, which were analyzed later in this study by NECAB1 CBA screening, showed the same autoantibody binding pattern as observed for patients 1–3, with exception of patient 7 (overview see Online Resource 2, Tab. 1). However, besides the characteristic NECAB1 pattern, additional antibody binding was observed with the serum and CSF from patient 2 (CSF: moderate staining of cerebellar Purkinje cells and molecular layer, Online Resource 1, Fig. 1a; serum: somatic staining of few neurons of the cerebellar granular cell layer, Online Resource 1, Fig. 1b) and patient 4 (staining of the cerebellar Purkinje cells, Online Resource 1, Fig. 1 d). In these cases, a second autoantibody type is suspected.

CBA with 35 different previously known brain antigens (Hu, Yo, Ri, CV2, PNMA2, ITPR1, Homer 3, CARP VIII, ARHGAP26, ZIC4, DNER/Tr, GAD65, recoverin, GABAB receptor, glycine receptor, DPPX, IgLON5, NMDAR, AMPAR, mGluR1, mGluR5, GluD2, LGI1, CASPR2, AQP4, MOG, ATP1A3, NCDN, flotillin1/2, KCNA2, AP3B2, Sez6L2, CNTN1, NF155 and NF186) revealed negative results for all patient sera.

### Identification of NECAB1 as target of the autoantibodies

Immunoprecipitation with rat hippocampus lysate and serum from patient 1 followed by SDS-PAGE revealed a protein band of approximately 40 kDa, which was identified as N-terminal EF-hand calcium-binding protein 1 (NECAB1, NCBI No. NP_071638.1) by mass spectrometry (Online Resource 1, Fig. 2a). Western blot with rat hippocampal lysate incubated with patient serum showed a reactive protein band of the same size. NECAB1 was also identified as target antigen of patient 4, while mass spectrometry of a protein band of the same size in the eluate fraction of patient 7 revealed cytoplasmic actin (Online Resource 1, Fig. 2b).

### Validation of autoantibodies by indirect immunofluorescence assay with recombinant NECAB1

**Fig. 2:**
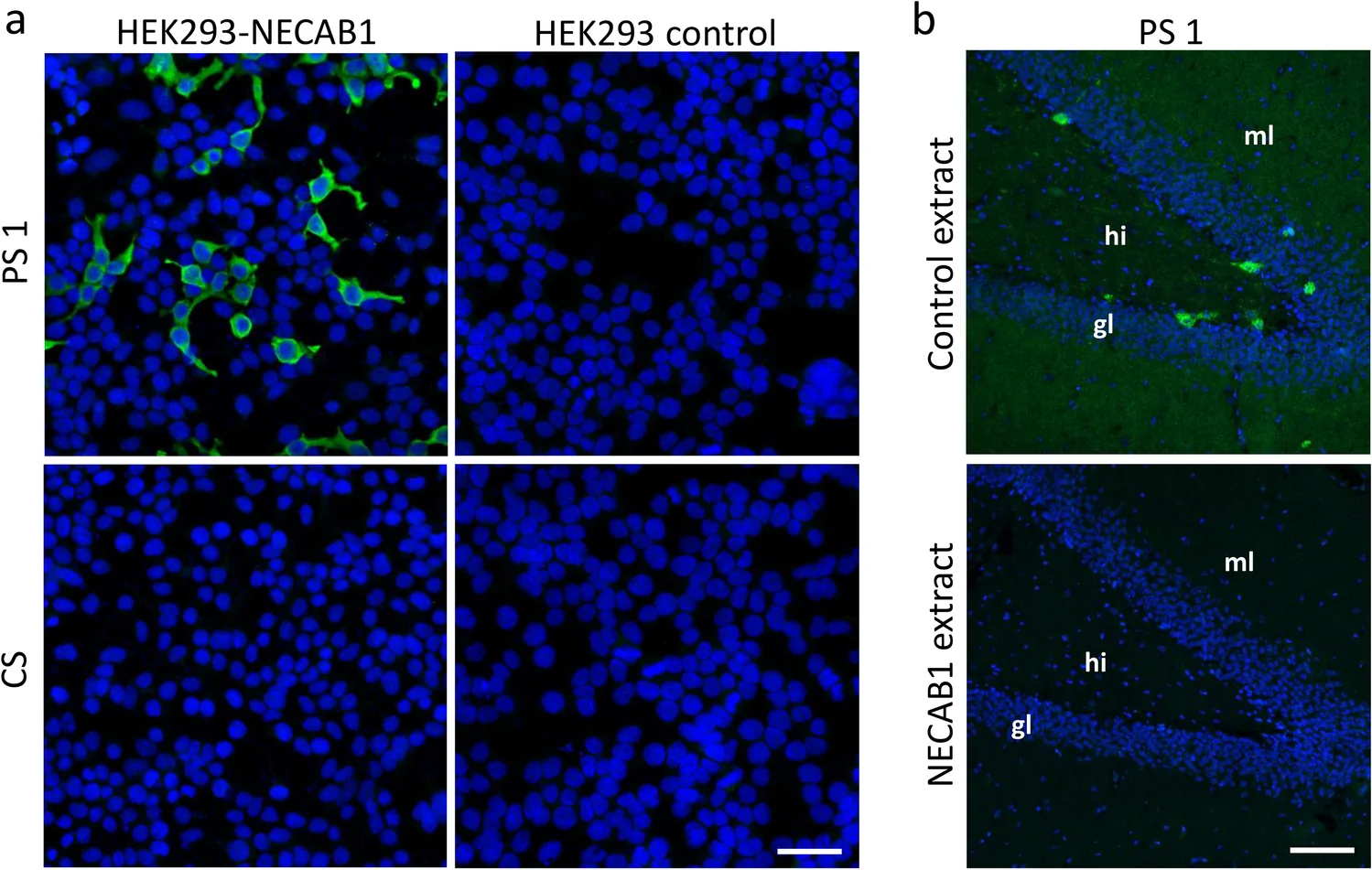
Immunofluorescence assay with patient serum and recombinant NECAB1. Cell based assay (CBA) with 1:100 diluted serum of patient 1 or control serum, incubated on acetone-fixed HEK293 cells expressing recombinant human histidine-tagged NECAB1 or empty vector transfected control cells (**a**). In a competitive tissue IIFA, 1:320 diluted serum of patient 1 was preincubated with 1:5 diluted recombinant human NECAB1 containing HEK293 cell extract or 1:5 diluted control extract one hour prior to incubation on rat hippocampal cryosections (**b**). In a second step, FITC-labeled (**a**) or Alexa Fluor 488-labeled (**b**) goat anti-human IgG was used. Cell nuclei were stained by TO-PRO-3 Iodide. Scale bars = 50 µm (**a**) or 100 µm (**b**). *hi* hilus, *gl* granula cell layer, *ml* molecular layer

In a competitive tissue IIFA using rat hippocampal sections, autoantibody binding was abolished by preincubation of diluted patient serum with HEK293 cell extract containing recombinant NECAB1 prior to incubation on neuronal tissue. However, it was unaffected by preincubation with a control HEK293 extract (Fig. 2b).

Recombinant cell-based indirect immunofluorescence assay (CBA) with HEK293 cells expressing human NECAB1 and 1:100 diluted sera from index patients (PS 1–3) showed autoantibody binding to acetone-fixed and thereby permeabilized HEK293-NECAB1 cells, which was not detected by incubation of empty vector transfected cells (Fig. 2a). Analysis of samples from 205 patients with suspected autoimmune disease revealed four additional anti-NECAB1 CBA-positive samples (PS 4–PS 7). However, tissue IIFA with rat cerebellar sections confirmed anti-NECAB1 autoantibodies only for three of four samples (PS 4–PS 6). NECAB1 CBA endpoint titers ranged from 1:320 to 1:32,000 in serum and from 1:100 to 1:3200 in CSF (for details see Table 1 and Online Resource 2, Tab. 1). Sera from 100 healthy donors (healthy controls, HC) and 303 sera or CSF samples from patients with different diseases (disease cohort, DC), including chronic inflammatory demyelinating polyneuropathy (CIDP, *n* = 33 sera), diabetic neuropathy (*n* = 30 sera), Guillain–Barré syndrome (GBS, *n* = 30 sera), small cell lung cancer (SCLC) without PNS (*n* = 49 sera), Alzheimer’s disease (*n* = 89 CSF), normal pressure hydrocephalus (*n* = 15 CSF), psychosis (*n* = 20 CSF), dementia with Lewy bodies (*n* = 12 CSF), and frontotemporal dementia (*n* = 25 CSF) did not show antibody binding to recombinant HEK293-NECAB1 cells by CBA.

### Validation of autoantibodies by Western blot with recombinant NECAB1 and patient sera

**Fig. 3:**
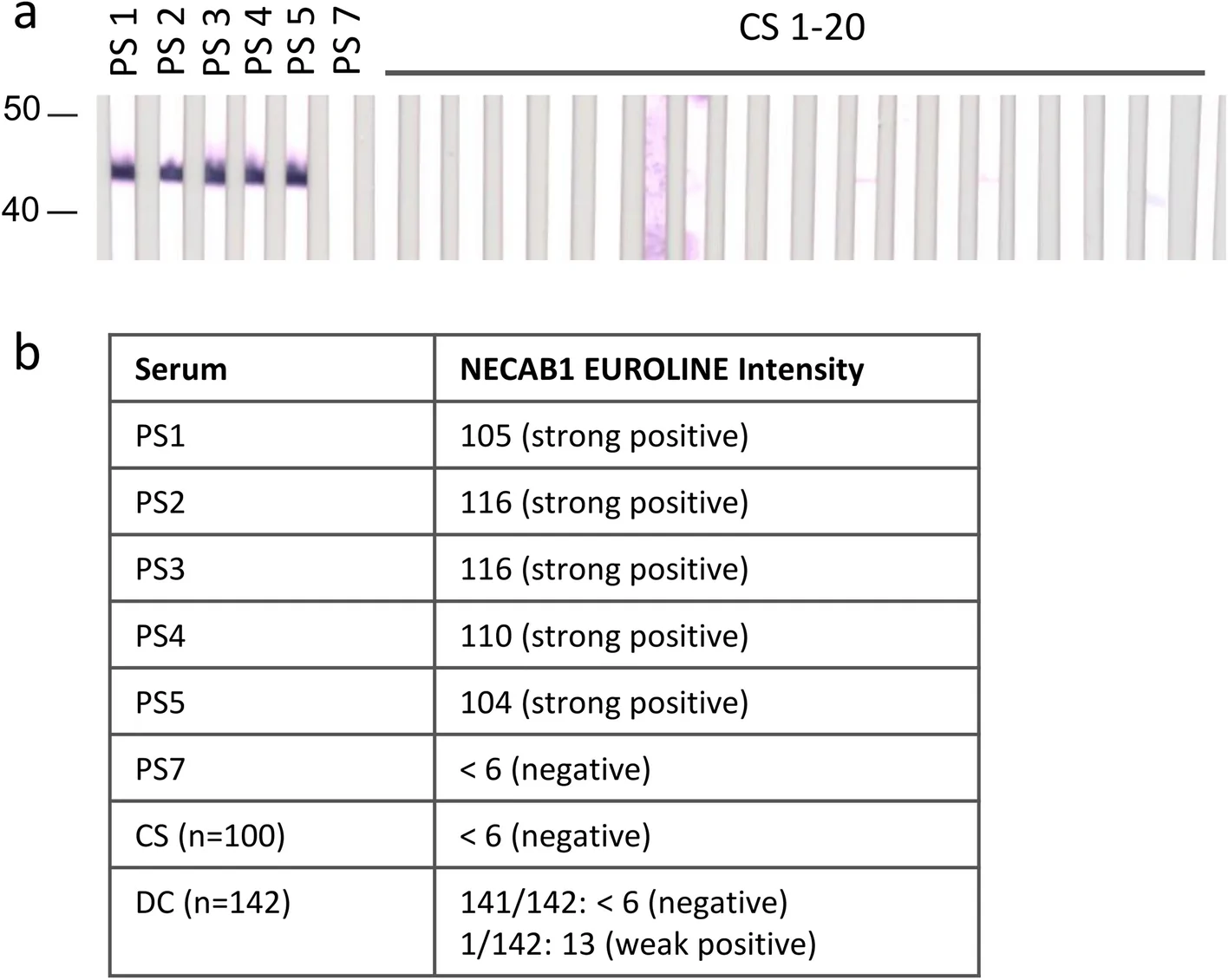
Immunoblot with purified recombinant human NECAB1. Western blot membranes (**a)** were incubated with 1:200 diluted patient sera (PS) or healthy control sera (CS). Results of line blot (**b)** with antigen coated membranes, 1:100 diluted patient sera (PS), healthy control sera (CS), and disease control sera (DC)

Western blot with recombinant human full-length NECAB1 purified from *E. coli* showed autoantibody binding with sera from patients 1–5. Serum from patient 7, and 20 healthy control sera (HC) showed no autoantibody binding to recombinant NECAB1 in Western blot (Fig. 3a and Online Resource 2, Tab. 1). PS 6 could not be analyzed due to limited sample amount.

### Validation of autoantibodies by line blot with recombinant NECAB1 and patient sera

Line blot with recombinant human full-length NECAB1 purified from *E. coli* revealed strong positive results with sera from patients 1–5, with intensity values between 104 and 116, while patient 7 and 100 healthy control sera were negative (intensity < 6) (Fig. 3b and Online Resource 2, Tab. 1). PS 6 could not be analyzed due to limited sample amount. Analysis of 142 disease control sera including chronic inflammatory demyelinating polyneuropathy (CIDP, *n* = 33 sera), diabetic neuropathy (*n* = 30 sera), Guillain-Barré syndrome (GBS, *n* = 30 sera), small cell lung cancer (SCLC) without PNS (*n* = 49 sera) revealed negative results in 141 of 142 cases, while one sample of the group SCLC without PNS was weak positive with a signal intensity value of 13 which is close to borderline (borderline signal intensities range from 6 to 10). The sample was NECAB1 CBA negative and did not show characteristic anti-NECAB1 binding pattern in a tissue immunofluorescence assay with rat hippocampal sections (data not shown). CSF samples of the disease control cohort (Alzheimer disease, normal pressure hydrocephalus, psychosis, dementia with Lewy bodies, and frontotemporal dementia) could not be analyzed by line blot due to limited sample amount.

## Discussion

In this study, we identified six patients with neurological autoimmune disease and cancer with autoantibodies against the N-terminal EF-hand calcium-binding protein 1 (NECAB1). NECAB1 is part of a family consisting of three proteins (NECAB1-3) that contain at least one EF-hand calcium-binding domain and are mainly expressed in neurons [12]. NECAB1 is localized in the cytoplasm of neurons and associates with synaptotagmin-1, a critical neuronal protein involved in membrane trafficking and synaptic vesicle exocytosis [13]. Calcium binding might induce a conformational change, which might lead to alteration of NECAB1 binding partners. Until now, not much is known about NECAB1 function, but in the context of NECAB1 gene mutations language disorders have been described, while schizophrenia and diabetes mellitus were associated with altered NECAB1 expression levels [12]. In mouse brain, NECAB1 is expressed in the dendrites of neurons, in the molecular layer of the dentate gyrus and, to a high extent, in the soma of hippocampal interneurons, where it is concentrated at the border between stratum radiatum and stratum lacunosum moleculare as well as in large cells of the hilar polymorphic layer [14]. The described expression pattern is consistent with our findings from neural rodent tissue IIFA (Fig. 1) and also matches the description and images from the recently first reported case with autoantibodies against NECAB1 [15], supporting the correlation of the observed patient IgG binding pattern observed in our study and NECAB1. Importantly, competitive inhibition experiments provided direct evidence for antigen specificity, as preincubation of patient samples with recombinant NECAB1-expressing HEK293 cell lysates completely abolished tissue staining (Fig. 2b). Future co-localization studies using validated anti-NECAB1 antibodies may further strengthen evidence for the correlation of the observed patient IgG staining pattern and NECAB1.

Furthermore, expression of NECAB1 and NECAB2 in dorsal root ganglia, including nociceptors, and spinal cord has been described [16]. Upregulation of NECAB1 expression in rat brain was shown in a temporal lobe epilepsy model [17], while it is downregulated in schizophrenia [18]. There is only limited information about NECAB1 expression in cancer so far. Analysis of colorectal cancer samples shows increased DNA methylation of the NECAB1 gene, leading to decreased NECAB1 gene expression. The overall survival was higher in patients with low NECAB1 gene methylation followed by increased expression [19]. Downregulation of NECAB1 expression was shown in more aggressive prostate tumors compared to indolent prostate tumors [20].

For both of our diagnostic cohorts, originating from two clinical immunologic laboratories in France and Germany, the numbers of all samples analyzed for neural autoantibodies in the time period from August 2019 to November 2024 were determined, as well as positive rates for the established PNS markers anti-Yo, anti-Tr/DNER, and anti-Ri and the encephalitis markers anti-NMDAR and anti-LGI1. Since no relevant differences in cohort composition were present, data of both cohorts were combined, increasing the number of evaluable samples to a total of 299,789 and thereby enhancing the interpretability of prevalence data. While the encephalitis markers were quite frequently positive (anti-NMDAR: 0.850%; anti-LGI1: 0.538%), the positive rates of the PNS markers were lower over both cohorts (anti-Yo: 0.104%; anti-Tr/DNER: 0.021%; anti-Ri: 0.034%), which is comparable with data from a retrospective laboratory-based cohort study of consecutive patients referred for neuronal autoantibody testing in a Spanish reference center (anti-NMDAR: 0.481%; anti-LGI1: 0.500%; anti-Yo: 0.084%; anti-Tr/DNER: 0.026%; anti-Ri: 0.026%) [21]. The frequency of the newly identified anti-NECAB1 autoantibodies (0.002%) is even lower with a positivity rate comparable to Neurexin-3a (0.006%, corresponding to one case) or DPPX (0.006%, corresponding to one case) [21]. However, additional cases may have been missed, as NECAB1 was not part of the routine screening panel during the study period.

The six anti-NECAB1-positive patients described in this study were all female at ages of 50–84 years. Across the cases with available clinical data (*n* = 5), cancer (lung and breast cancer) was diagnosed beside the neurological symptoms in all individuals, consistent with a phenotype of PNS. Despite its low detection frequency, anti-NECAB1 represents a highly informative biomarker. The observed 100% tumor association in our cohort underscores its relevance for cancer screening and clinical management. Identification of these patients may facilitate earlier tumor detection while simultaneously expanding knowledge regarding disease course and responsiveness to immunotherapy.

While direct analysis of NECAB1 expression in tumor tissue was not possible, the concurrence between autoantibody positivity and oncologic disease supports a paraneoplastic mechanism. The intracellular localization of NECAB1 and the consistent recognition of denatured NECAB1 by all patient sera on Western blot suggest that NECAB1 shares key features with classical onconeural antigens, including Hu, Yo, and Ri, which are established high-risk markers of paraneoplastic neurological syndromes [22–24].

The majority of the anti-NECAB1-positive patients with available clinical data suffered from limbic encephalitis with memory disturbance, neuropsychiatric, behavioral and cognitive symptoms. Apart from this, one patient (P2) suffered from cerebellar ataxia with cognitive decline and one from sensory-motor neuronopathy (P4). With this limited number of patients, anti-NECAB1 autoantibodies cannot yet be linked to one single neurological syndrome. However, it is worth mentioning that PS2 and PS4 showed additional binding patterns in neuronal tissue IIFA, indicating that additional autoantibodies may contribute to the clinical manifestations in these patients. Neuronal tissue IIFA using rat brain sections and CSF from patient 2 showed autoantibody binding to cerebellar Purkinje cells, while IIFA with mouse brain and serum from patient 2 showed staining of a subset of cerebellar granular layer neurons. This patient presented with cerebellar syndrome with cognitive decline rather than limbic encephalitis. These observations raise the possibility that additional autoantibodies may contribute to the clinical manifestations in some patients.

In patient 4, who had sensory-motor neuronopathy and lung cancer with parieto-occipital metastasis, intracranial disease may have contributed to or interfered with neurological symptoms. The presence of additional Purkinje cell staining suggests a potential second autoantibody in this patient’s serum, which could partially contribute to the divergent neurological phenotype. However, as NECAB1 expression was also described in dorsal root ganglia [16], a direct link between anti-NECAB1 autoimmunity and sensory-motor neuronopathy is also conceivable.

During the preparation of the manuscript, one anti-NECAB1-positive patient (F, 75) was described [15]. The neurological phenotype of this patient, suffering from encephalitis with behavioral changes, status epilepticus, and right upper limb posturing, is in line with the most frequent findings in our case series. However, cancer was not detected nor excluded with certainty in this case. As in the cases included in this study, the patient reported was female and anti-NECAB1 IgG was found in serum and CSF.

All cases with available cerebrospinal fluid (CSF) data (P1, 2, 4, 5, 6) demonstrated high anti-NECAB1 titers by CBA in CSF (1:100 to 1:3200), and four patients (P2, P4, P5, and P6) showed elevated numbers of white blood cells (12–36 cells/mm^3^), indicating that inflammatory CSF changes might contribute to the CNS pathology. An exception was patient 3, with a 1:10 CSF titer, while the serum titer reached 1:3200. Clinical information for this patient was not available, limiting interpretation.

Serum from patient 7 showed autoantibody binding to recombinant NECAB1-expressing cells (titer 1:1000) but did not bind hippocampal interneurons in tissue IIFA. Western blot analysis using purified recombinant NECAB1 as well as line blot with the same antigen demonstrated strong autoantibody binding for sera from patients 1–5, while serum from patient 7 did not show any binding. For further clarification, Western blot and immunoprecipitation with rat hippocampal lysate were performed with serum from patient 7 and patient 4. While a reactive band was detected in the size of NECAB1, and NECAB1 was identified by mass spectrometry with serum from patient 4, there was no reactive band for patient 7, and mass spectrometry of a band of the respective size revealed cytoplasmic actin (Online Resource 1, Fig. 2b), demonstrating that NECAB1 is not the target antigen in the case of patient 7. Together with the male sex and lack of known cancer, this case most likely represents a non-specific autoantibody binding in the CBA. The notion that the NECAB1 cell-based assay can yield false-positive results suggests line blot as the recommended screening method and highlights the value of confirmation by independent methods such as tissue IIFA.

Overall, the NECAB1 cell-based assay showed a high specificity, with no unspecific reactions in 100 healthy controls and 303 disease controls at a 1:100 (sera) or 1:10 (CSF) dilution. Nevertheless, immunoblotting with purified antigen seems to enhance the specificity, probably by excluding potential cross-reactivity to other proteins within the cell-based system. Conversely, one sample of the disease control cohort showed a weak positive reaction in the line blot assay. Especially in this subgroup (SCLC without PNS) of the disease control cohort, neither the presence of low-titer antibodies against NECAB1 in the patient sample nor the subsequent development of neurological symptoms in the patient with SCLC can be excluded. However, confirmatory testing by tissue IIFA provides the highest reliability for results generated with a monospecific recombinant protein assay.

The data suggest that anti-NECAB1 antibodies are associated with cancer and neurological symptoms in this cohort, reinforcing the potential utility of anti-NECAB1 testing in the serological workup for suspected PNS, particularly when autoimmune encephalitis is considered. According to Graus et al. [10] it is likely that NECAB1 autoantibodies will be classified as high-risk oncological antibodies in the future, with > 70% of cases associated with cancer and clinical presentations matching the criteria of high-risk neurological phenotypes. Future studies with higher numbers of cases are needed to further unravel their clinical significance.

Upon anti-NECAB1 positivity in a patient sample, a targeted tumor screen should be considered if a malignancy is not already known, given the paraneoplastic association observed in this study. To avoid the risk of false-positive results with monospecific assays confirmatory testing by a second independent method, preferably tissue IIFA, is reasonable.

One limitation of this study is the relatively small number of patients, especially those with detailed clinical information. Potential presence of additional antibodies in two patients complicates the elucidation of the clinical phenotype. Lack of tumor tissue availability is the missing link between malignancy and autoantibodies. Analysis of tumor specimens from anti-NECAB1-positive PNS patients in future studies would be of interest, as there is limited information about NECAB1 expression in cancer so far.

## Conclusions

Autoantibodies against NECAB1 appear to be linked with cancer and neurological symptoms in this cohort, demonstrating their potential role as biomarkers in suspected PNS and autoimmune encephalitis. Based on these findings anti-NECAB1 testing could be integrated into serological panels for PNS workups, especially when autoimmune encephalitis is suspected, and tumor screening should be considered upon detection.

## Electronic supplementary material

Below is the link to the electronic supplementary material.

Supplementary file1 (PDF 334 KB)

Supplementary file2 (PDF 136 KB)

## Data Availability

All data generated or analyzed during this study are included in this published article.

## Appendix

### Case reports

#### Patient 2

A 65-year-old woman with a history of left-sided breast cancer presented with a subacute, progressive cerebellar syndrome in the setting of newly diagnosed contralateral breast cancer. She initially developed gait instability, dysarthria, oscillopsia, vertigo, intermittent diplopia, and neck pain. Neurological examination consistently demonstrated cerebellar signs, including gaze-evoked nystagmus, dysarthria, limb and truncal ataxia, and a broad-based, insecure gait that later required a walker. At the peak of the disease, the patient was unable to stand or walk. Motor strength, sensory examination, and reflexes were normal, and cognition was preserved apart from mild psychomotor slowing.

Initial brain MRI showed no ischemic, inflammatory, metastatic lesions, or cerebellar atrophy. Repeated contrast-enhanced MRIs revealed persistent supratentorial and infratentorial meningeal contrast enhancement without nodular lesions or parenchymal involvement, remaining stable over time. Although suspicious, these findings were not diagnostic of meningeosis carcinomatosa. No spinal cord pathology or osteal metastases were detected by cervical spine MRI. CSF analysis repeatedly demonstrated pleocytosis and markedly elevated protein levels, while cytology for malignant cells remained inconclusive. Extensive paraneoplastic and neuropil antibody panels were initially negative, but subsequent testing identified IgG autoantibodies against NECAB1, supporting an autoimmune paraneoplastic etiology. CSF analysis four months after symptom onset revealed oligoclonal bands type 2, indicating intrathecal autoantibody synthesis. Neurophysiological examination and cerebrovascular ultrasound were unremarkable.

Oncological evaluation revealed a multiseptated right breast tumor with pathologically enlarged right axillary lymph nodes on CT. Mammography confirmed multifocal right-sided breast cancer (BI-RADS 5) with axillary metastases, while the left breast showed no recurrence. Histology demonstrated an invasive multifocal carcinoma of no special type, hormone receptor–positive (ER 100%, PgR 70–80%), HER2neu 1+, with variable Ki-67 indices. The patient had previously been treated for left-sided hormone receptor–positive breast cancer with surgery, chemotherapy, radiotherapy, and adjuvant aromatase inhibitor therapy.

Given the progressive cerebellar syndrome, inflammatory CSF findings, neuronal autoantibodies, and newly diagnosed metastatic breast cancer, a diagnosis of paraneoplastic cerebellitis was made, with suspected meningeosis carcinomatosa remaining a challenging differential diagnosis. At last follow-up, 38 months after symptom onset, the patient was stable under chemotherapy. She had mild gait disturbances but was able to walk longer distances using a walker.

#### Patient 4

A 62-year-old woman presented with a subacute onset of progressive dysphagia over several months, accompanied by unintentional weight loss. Upon admission, neurological examination revealed a sensory deficit and paresthesia in a glove-and-stocking distribution, proprioceptive impairment in the right limb, right-predominant dysmetria, and gait instability characterized by a significantly widened base of support. Her modified Rankin Scale (mRS) score was 3. A thoracoabdominopelvic CT scan and PET-CT revealed parahilar lesions, a large colonic mass with peritoneal invasion, as well as pelvic and mediastinal lymphadenopathy, the latter partially compressing the esophagus. Brain MRI demonstrated a parieto-occipital metastasis, while cervical spine MRI showed no significant abnormalities. Histological analysis confirmed pulmonary adenocarcinoma as the primary source. Electroneurography revealed features compatible with sensorimotor demyelinating neuropathy. CSF analysis showed 12 white blood cells/mm^3^, elevated protein levels (0.81 g/L), and the presence of mirrored oligoclonal bands (type 4). Immunofluorescence revealed atypical anti-neuronal staining. However, no specific antibodies were detected in CSF or serum at that time. Treatment was initiated 18 days after symptom onset and included intravenous methylprednisolone 120 mg/day, followed by an oral tapering schedule and chemotherapy (carboplatin and paclitaxel). Despite treatment, the patient’s oncological condition progressed (peritoneal carcinomatosis) and she died by cancer-related complications 4 months after symptom onset (anemia requiring transfusions, and abdominal sepsis).

#### Patient 5

An 84-year-old woman presented with subacute onset of memory impairment, anorexia, and unintentional weight loss. Over the subsequent 3 weeks, she developed apathy, headache, pruritus, and hypersomnia. She was therefore admitted for further evaluation, where neurological examination revealed spatial–temporal disorientation and psychomotor slowing, with a Mini Mental State Examination score of 8/30 and a mRS score of 3. Laboratory tests revealed hyponatremia (126 mmol/L), hypomagnesemia (0.58 mmol/L), and hypokalemia, all corrected with oral supplementation. Brain MRI showed bilateral temporal FLAIR hyperintensities. CSF analysis demonstrated 16 white cells/mm^3^ and elevated protein levels (0.97 g/L). Immunofluorescence demonstrated hippocampal staining. No specific antibody was identified at the time of testing. Tumor screening subsequently revealed a non-keratinizing epidermoid carcinoma of the lung with nodal metastases. Given the clinical and paraclinical findings suggestive of limbic encephalitis, intravenous immunoglobulins (IVIG) were initiated. The patient received four courses of IVIG (0.4 g/kg/day) over the next 4 months. Regarding the oncological management, the tumor was surgically resected, followed by chemotherapy (carboplatin and paclitaxel) and mediastinal radiotherapy, resulting in a favorable oncological response. Despite the initial treatment, the patient continued to experience cognitive deficits and spatial disorientation, requiring substantial family support. Retrospective analysis of cerebrospinal fluid demonstrated NECAB1-specific reactivity in a cell-based assay. As a second-line therapy, rituximab was introduced, resulting in a gradual improvement over the following months. At her most recent evaluation, after 69 months of follow-up, she only had mild temporal disorientation and required assistance for medication management. No clinical relapses or recurrence of the tumor were detected.

#### Patient 6

A previously healthy 50-year-old female presented to the emergency department with generalized convulsions. She was subsequently admitted for further evaluation. MRI showed bilateral temporal FLAIR hyperintensities, more pronounced on the right. Cerebrospinal fluid analysis revealed 36 white cells/mm^3^, the presence of oligoclonal bands and immunofluorescence staining showing granular labeling in the cerebellar cortex and cerebellar white matter, with marked immunoreactivity in the CA3 region of the hippocampus. No specific antibodies were identified at that time. Cancer screening revealed a 5 mm solitary lung nodule and invasive ductal carcinoma of the breast (grade III, Ro+, Ki-67 50%), for which she underwent surgery, radiotherapy, chemotherapy, and hormonal therapy with anastrozole. Based on the suspected diagnosis of paraneoplastic limbic encephalitis, she received six courses of cyclophosphamide. Clinical stabilization and good seizure control was achieved under concurrent anti-seizure medication. Approximately 1 year after the last cyclophosphamide dose, she developed paranoid delusions and subsequently progressed until hospital readmission 2 years later due to acute psychiatric decompensation, presenting with visual and auditory hallucinations and persecutory delusions. Neurological examination was unremarkable, except for memory and attention deficits and reasoning difficulties (Montreal Cognitive Assessment score 17/30; modified Rankin Scale [mRS] score was 3). Follow-up MRI revealed hippocampal atrophy, EEG did not reveal any significant alterations, while oncological reassessment showed no recurrence of the breast cancer and resolution of the lung nodule. CSF analysis showed oligoclonal bands (without proteinorrhachia or pleocytosis) and immunohistochemistry showed immunoreactivity in hippocampal interneurons (especially in the hilus), thalamus and colliculus, with milder staining in the molecular layer of the dentate gyrus. In these areas, the patient’s CSF strongly labeled soma and dendrite of isolated neurons. She received neuroleptic treatment with no additional immunotherapy. She was discharged and, at the last follow-up (41 months), her modified Rankin Scale score had improved to 2. Retrospective testing of cerebrospinal fluid obtained from the second lumbar puncture yielded a positive result for NECAB1 antibodies. Further immunotherapy options are currently being considered.
