# Supplementary material for: Autoantibodies against N-terminal EF-hand calcium-binding protein 1 in neurological syndromes and cancer
Source: J Neurol. 2026 Nov 14;273(12):717. doi: 10.1007/s00415-026-14257-3 (PMC13769751; doi:10.1007/s00415-026-14257-3)
Supplement: Supplementary file 1 [file 415_2026_14257_MOESM1_ESM.pdf]

Autoantibodies against N-terminal EF-hand calcium-binding protein 1 in neurological syndromes and cancer

Madeleine Scharf et al.

Journal of Neurology

Corresponding authors:

madeleine.scharf@revvity.com; jerome.honnorat@chu-lyon.fr

Online Resource 1

Supplementary Figures

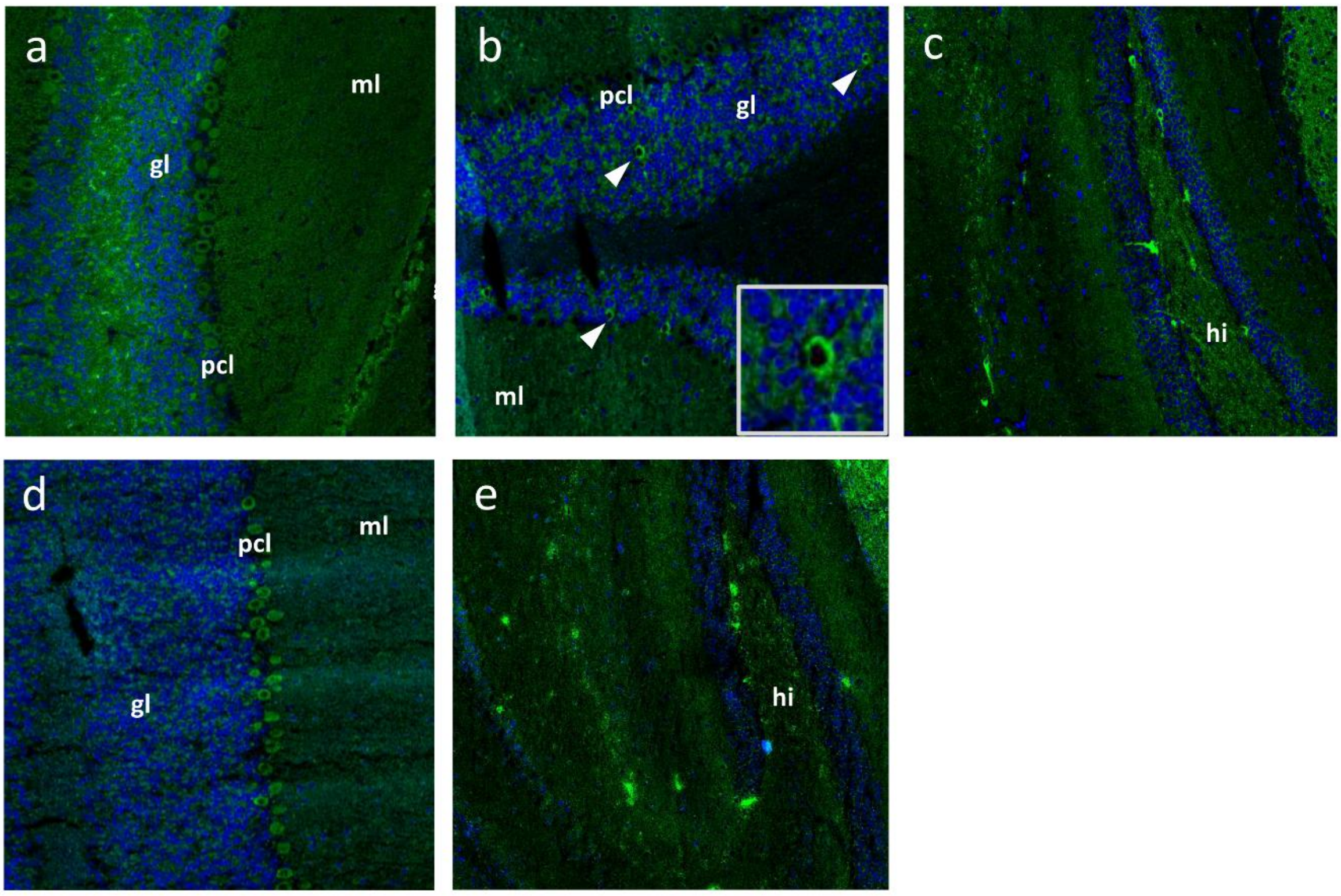

Online Resource 1: Figure 1 Anti-NECAB1 and additional tissue IIFA reactivities of P2 and P4. 1:10 diluted CSF from patient 2 shows binding to Purkinje cells and white

matter of rat cerebellum (a). 1:1000 diluted serum from patient 2 shows cytoplasmic staining of few neurons (arrowheads) of the granular cell layer of mouse cerebellum (b, inlay shows reactive neuron, enlarged) and staining of hippocampal interneurons (c). 1:100 diluted serum from patient 4 shows binding to Purkinje cells of mouse cerebellum (d) and to hippocampal interneurons (e). gl: granular cell layer; pcl: Purkinje cell layer; ml: molecular layer; hi: hilus

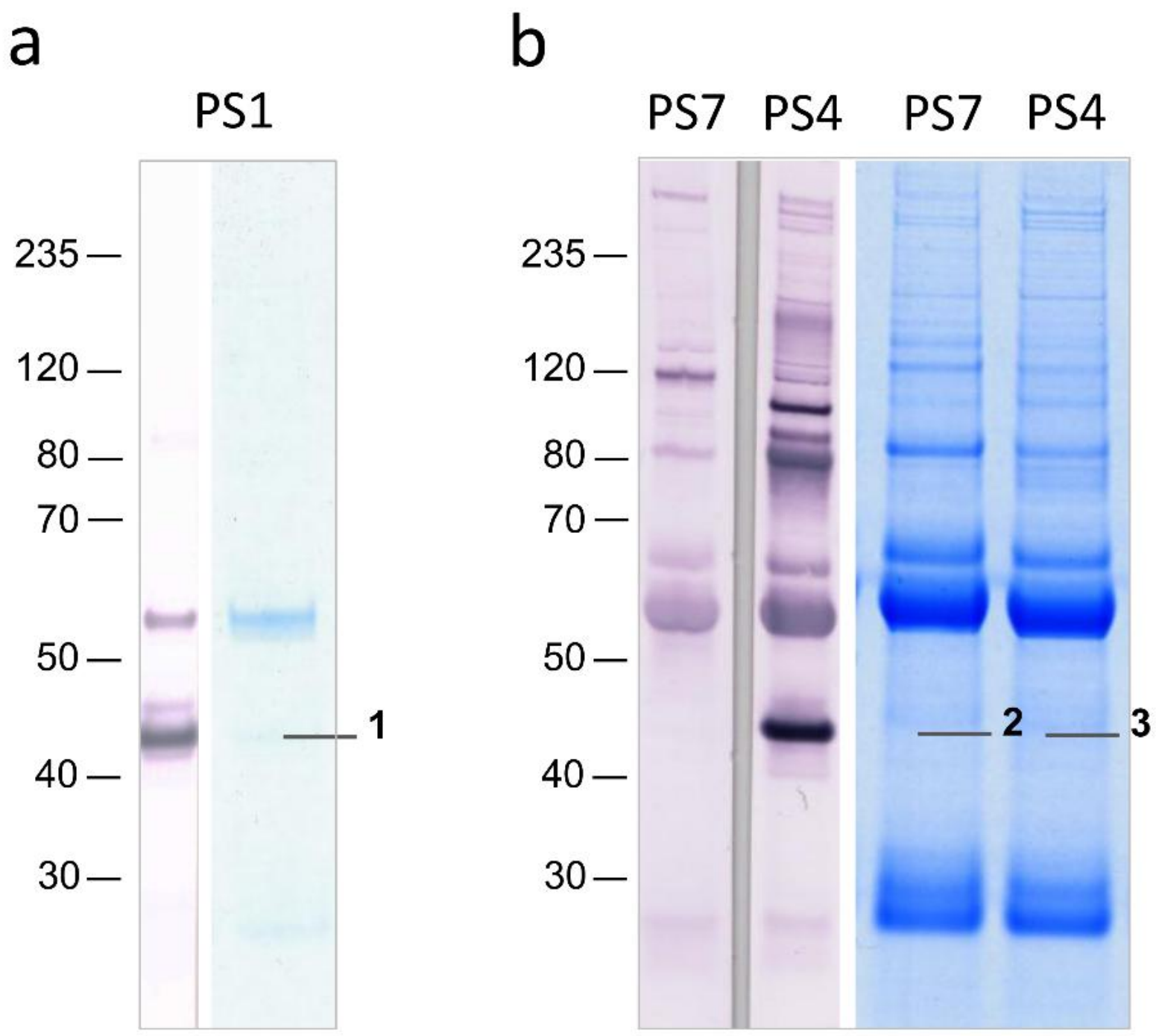

Online Resource 1: Figure 2 Western blot (WB) and immunoprecipitation (IP) with rat hippocampal lysate and patient sera. Bound proteins were separated by SDS-PAGE and stained with Coomassie. Protein bands analyzed by mass spectrometry are indicated. (a) IP with serum from patient 1, protein band 1 was identified as N-terminal EF-hand calcium-binding protein 1 (NECAB1). (b) IP with serum from patient 4 and patient 7, protein band 2 was identified as cytoplasmic actin, protein band 3 was identified as NECAB1
