# Supplementary material for: Autoantibodies against N-terminal EF-hand calcium-binding protein 1 in neurological syndromes and cancer
Source: J Neurol. 2026 Nov 14;273(12):717. doi: 10.1007/s00415-026-14257-3 (PMC13769751; doi:10.1007/s00415-026-14257-3)
Supplement: Supplementary file 2 [file 415_2026_14257_MOESM2_ESM.pdf]

Autoantibodies against N-terminal EF-hand calcium-binding protein 1 in neurological syndromes and cancer

Madeleine Scharf et al.

Journal of Neurology

Corresponding authors:

madeleine.scharf@revvity.com; jerome.honnorat@chu-lyon.fr

Online Resource 2

Supplementary Table

Online Resource 2: Table 1 Overview of results from neuronal tissue IIFA, NECAB1 CBA, NECAB1 Western Blot, and NECAB1 line blot.

Patient	Sample type	Neuronal Tissue IIFA	NECAB1 CBA endpoint titer	NECAB1 Western Blot signal intensity [a]	NECAB1 Lineblot signal intensity score [b]
P1	Serum	Anti-NECAB1 typical	1:3,200	+++	105
	CSF	Anti-NECAB1 typical	1:1,000	N/A	N/A
P2	Serum	Anti-NECAB1 typical	1:10,000	+++	116
	CSF	Anti-NECAB1 typical	1:320	N/A	N/A
P3	Serum	Anti-NECAB1 typical	1:10,000	+++	116
	CSF	Anti-NECAB1 typical	1:10	N/A	N/A
P4	Serum	Anti-NECAB1 typical	1:320	+++	110
	CSF	Anti-NECAB1 typical	1:320	N/A	N/A
P5	Serum	Anti-NECAB1 typical	1:32,000	+++	104
	CSF	Anti-NECAB1 typical	1:3,200	N/A	N/A
P6	Serum [c]	N/A	N/A	N/A	N/A
	CSF	Anti-NECAB1 typical	1:100	N/A	N/A
P7	Serum	atypical	1:1,000	negative	< 6
	CSF	atypical	negative	N/A	N/A

[a] determined by visual inspection; [b] determined by EUROLineScan software; [c] Sample not available (N/A). NECAB1 CBA: serum cut off ≥ 1:100 and CSF cut off ≥ 1:10; NECAB1 Western Blot: serum dilution 1:200, signal intensity from “+” to “+++” = positive; NECAB1 line blot: serum dilution 1:100, cut off > 10

Online Resource 2: Table 2 Time of sample collection combined with demographical data and main clinical and serological findings

**Patient #, sex, age at neurological onset**	**Main clinical features**	**Cancer**	**NECAB1 IgG CBA**	**Time from symptom onset to sampling (months)**	**Time from tumor detection to sampling (months)**
#1<br>female, 53 y	Limbic encephalitis	Breast cancer	Serum 1:3.200<br>CSF 1:1.000	Serum: N/A<br>CSF: N/A	Serum: N/A<br>CSF: N/A
#2<br>female, 65 y	Cerebellar syndrome with cognitive decline (ataxia, dysarthria, double vision, vertigo)	Breast cancer	Serum 1:10.000<br>CSF 1:320	Serum: 3<br>CSF: 3	Serum: 0<br>CSF: 0
#4<br>female, 62 y	Sensory-motor neuronopathy (dysphagia, unintentional weight loss, paresthesia and equilibrium deficits)	Lung cancer<br>(metastatic pulmonary adenocarcinoma, including a right parietooccipital brain metastasis)	Serum 1:320<br>CSF 1:320	Serum: 2<br>CSF: N/A	Serum: 2<br>CSF: N/A
#5<br>female, 84 y	Limbic encephalitis (memory disturbance, anorexia, weight loss, apathy, headache, pruritus, sleepiness)	Lung cancer<br>(non keratinizing epidermoid cancer with PDL-1 expression; 2 lymph node metastasis)	Serum 1:32.000<br>CSF 1:3.200	Serum: 3<br>CSF: 3	Serum: -2<br>CSF: -2
#6<br>female, 50 y	Limbic encephalitis (generalized seizures, visual and auditive hallucinations, persecutory delusions, memory and executive disturbances)	Breast cancer<br>(IDC grade III Ro+, Ki 50%; 5 mm solitary lung nodule)	Serum N/A<br>CSF: 1:100	Serum: N/A<br>CSF: N/A	Serum: N/A<br>CSF: N/A
